# New tools for carbohydrate sulfation analysis: heparan sulfate 2-*O*-sulfotransferase (HS2ST) is a target for small-molecule protein kinase inhibitors

**DOI:** 10.1042/BCJ20180265

**Published:** 2018-08-14

**Authors:** Dominic P. Byrne, Yong Li, Krithika Ramakrishnan, Igor L. Barsukov, Edwin A. Yates, Claire E. Eyers, Dulcé Papy-Garcia, Sandrine Chantepie, Vijayakanth Pagadala, Jian Liu, Carrow Wells, David H. Drewry, William J. Zuercher, Neil G. Berry, David G. Fernig, Patrick A. Eyers

**Affiliations:** 1 Department of Biochemistry, Institute of Integrative Biology, University of Liverpool, L69 7ZB Liverpool, U.K.; 2 Centre for Proteome Research, Institute of Integrative Biology, University of Liverpool, L69 7ZB Liverpool, U.K.; 3 Laboratory CRRET CNRS 9215, Université Paris-Est, CRRET (EA 4397/ERL CNRS 9215), UPEC, F-94010 Créteil, France; 4 Glycan Therapeutics, 617 Hutton Street, Raleigh, NC 27606, U.S.A.; 5 UNC Eshelman School of Pharmacy, University of North Carolina at Chapel Hill, Chapel Hill, NC 27599, U.S.A.; 6 Structural Genomics Consortium, UNC Eshelman School of Pharmacy, University of North Carolina at Chapel Hill, Chapel Hill, NC 27599, U.S.A.; 7 Lineberger Comprehensive Cancer Center, University of North Carolina at Chapel Hill, Chapel Hill, NC 27599, U.S.A.; 8 Department of Chemistry, University of Liverpool, L69 7ZD Liverpool, U.K.

**Keywords:** heparan sulfate, heparin, inhibitor, kinase, sulfotransferase

## Abstract

Sulfation of carbohydrate residues occurs on a variety of glycans destined for secretion, and this modification is essential for efficient matrix-based signal transduction. Heparan sulfate (HS) glycosaminoglycans control physiological functions ranging from blood coagulation to cell proliferation. HS biosynthesis involves membrane-bound Golgi sulfotransferases, including HS 2-*O*-sulfotransferase (HS2ST), which transfers sulfate from the cofactor PAPS (3′-phosphoadenosine 5′-phosphosulfate) to the 2-*O* position of α-l-iduronate in the maturing polysaccharide chain. The current lack of simple non-radioactive enzyme assays that can be used to quantify the levels of carbohydrate sulfation hampers kinetic analysis of this process and the discovery of HS2ST inhibitors. In the present paper, we describe a new procedure for thermal shift analysis of purified HS2ST. Using this approach, we quantify HS2ST-catalysed oligosaccharide sulfation using a novel synthetic fluorescent substrate and screen the Published Kinase Inhibitor Set, to evaluate compounds that inhibit catalysis. We report the susceptibility of HS2ST to a variety of cell-permeable compounds *in vitro*, including polyanionic polar molecules, the protein kinase inhibitor rottlerin and oxindole-based RAF kinase inhibitors. In a related study, published back-to-back with the present study, we demonstrated that tyrosyl protein sulfotranferases are also inhibited by a variety of protein kinase inhibitors. We propose that appropriately validated small-molecule compounds could become new tools for rapid inhibition of glycan (and protein) sulfation in cells, and that protein kinase inhibitors might be repurposed or redesigned for the specific inhibition of HS2ST.

## Introduction

Biological sulfation is a widespread reversible covalent modification found throughout nature [1]. The regulated sulfation of saccharides is critical for cellular signalling, including regulatory interactions between extracellular glycoproteins that control signal transduction and high-affinity interactions between different cellular surfaces [2]. In addition to providing mechanical strength, the sulfate-rich extracellular matrix also represents a hub for sulfation-based communication through growth factor signalling [3]. For example, FGF–receptor interactions and intracellular signalling to the ERK pathway are blunted in the absence of appropriate 2-O sulfation driven by heparan sulfate (HS)-modifying enzymes [4–9], while sulfation of the tetrasaccharide Sialyl Lewis^X^ antigen on glycolipids controls leukocyte adhesion to the endothelium during inflammation [10,11]. Inappropriate glycan sulfation can therefore underlie aspects of abnormal signalling, infection, inflammation and, increasingly, human neuropathies [12], suggesting that targeting of carbohydrate sulfation dynamics using small-molecule enzyme inhibitors may be of value in both basic and translational research [13]. Indeed, the current limited chemical toolbox to rapidly modify and study glycan sulfation is based around small-molecule inhibitors of sulfatase-2 (Sulf-2), such as OKN-007 [14] or heparanase inhibitors and HS mimics, including roneparstat and PG545, which have been employed for basic and clinical investigation [15].

Glycan sulfotransferases (STs) can be classified into several families depending on the positional substrate specificity of enzymes for their respective sugar substrates [16,17]. HS 2*-O*-sulfotransferase (HS2ST) is required for the generation of HS, which is an abundant unbranched extracellular glycosaminoglycan with key roles in a range of physiological functions, most notably growth factor-dependent signalling related to development, cell migration and inflammation [18]. HS2ST is a transmembrane protein whose catalytic domain faces into the lumen of the Golgi compartment, and catalyses the sulfation of iduronic acid and, to a lesser extent β-d-glucouronate (GlcA), during the enzymatic assembly of secretory HS proteoglycans [18,19]. HS2ST transfers the sulfo moiety from PAPS (3′-phosphoadenosine 5′-phosphosulfate) sulfate donor to the C2 hydroxyl of IdoA that lies adjacent to an *N*-sulfated glucosamine residue, generating a 2-*O*-sulfated saccharide unit [20–22]. Removal of the sulfate by endosulfatases such as Sulf-2, or more general HS processing by heparanase, also contributes to the complex physiological patterns of carbohydrate editing found *in vivo* [23].

The analysis of murine models lacking HS2ST reveals central roles for 2-*O*-sulfated HS in kidney development and neuronal function, and for signalling through WNT- and FGF-dependent pathways [8,18,24–26]. However, in order to carefully control and examine the dynamics and structural heterogeneity of 2-O sulfation patterns in HS, which are the consequences of nontemplate-based synthesis of HS and complex dynamic sulfation patterns, new small-molecule approaches for the direct, reversible inhibition of sulfotransferase enzymes are urgently required. In particular, these need to be deployed using chemical biology strategies to overcome deficiencies associated with genetic disruption approaches relevant to development and/or compensatory glycosylation or signalling mechanisms [27].

Mechanistic parallels between the enzymatic pathway of biological sulfation by sulfotransferases [28] and phosphorylation by protein kinases [29] are apparent since both enzyme classes transfer charged chemical units from an adenine-based nucleotide cofactor to a (usually) polymeric acceptor structure. The biological analysis of protein kinases, which are thought to employ a similar ‘in-line’ enzyme reaction as the 2-*O-*sulfotransferases [28] when transferring phosphate to peptide targets [30], has been revolutionised by the synthesis and wide availability of small-molecule inhibitors [31]. Many of these compounds were originally discovered in screens with ATP-competitive inhibitor libraries using oncology-associated target enzymes [32]. Protein kinases have proved to be exceptional targets for the development of therapeutic agents in humans, and ∼50 kinase inhibitors have been approved, or will soon be approved, for cancer and anti-inflammatory indications [33]. To help diversify and accelerate this process, validated open-source panels of such inhibitors, such as the Public Kinase Inhibitor Set (PKIS), have been assembled for screening purposes, constituting a variety of chemotypes for unbiased small-molecule inhibitor discovery, which can be applied to a diverse range of protein targets [34].

The analysis of carbohydrate sulfation currently relies heavily on genetic, biophysical (NMR) and combinatorial organic chemistry and enzymatic analysis, with only a handful of low-affinity inhibitors of carbohydrate sulfotransferases ever having been disclosed [13,35]. More recently, a relatively potent inhibitor of the related type IV aryl sulfotransferase [36] and much lower affinity oestrogen sulfotransferase inhibitors [37–39] were reported. Owing to a lack of any selective chemical tool compounds, cellular glycan sulfation remains understudied, relying on non-specific cellular methods such as chlorate exposure [40], and the field remains ripe for technological innovation and new chemical biology approaches. Early attempts to discover such molecules among small, relatively unfocussed, kinase-based libraries led to the discovery of low-affinity purine and tyrphostin-based inhibitory compounds, which are well-established chemical classes of protein kinase inhibitor [35]. This raises the question as to whether PAPS-dependent sulfotransferases are general inhibitory targets for new or repurposed small molecules that target nucleotide-binding sites, especially broader families of compounds originally developed as protein kinase inhibitors. However, the low throughput nature of radioactive (^35^S-PAPS) TLC or HPLC-based assays typically used for sulfotransferase analysis [35,41,42], and the relatively low potency of current hits, argues for new approaches to assay and screen more diverse selections of focused or larger chemical libraries.

In the present paper, and in a related study employing tyrosyl protein sulfotranserases [43], we describe novel *in vitro* methods for assaying recombinant HS2ST, one of which employs a fluorescent-based detection system with a hexasaccharide substrate. PAPS-dependent sulfation of the substrate at the 2-O position of the IdoA residue leads to a change in substrate chemical properties, which can be detected as a real-time mobility shift in a high-throughput microfluidic assay format originally developed for the analysis of peptide phosphorylation [44,45]. We exploit this assay alongside differential scanning fluorimetry (DSF) to screen a small-molecule PKIS library, characterising HS2ST susceptibility towards a variety of cell-permeable compounds. We propose that appropriately validated small-molecule ligands might become invaluable probes for rapid cellular inhibition of HS2STs, and that further iteration could lead to the discovery and synthesis (or repurposing) of small molecules, including compound classes currently employed as kinase inhibitors, to probe cellular HS2ST function.

## Experimental

### Materials and methods

#### Chemicals and compounds

Porcine intestinal heparin was from Sigma, oligomeric saccharide standards, termed dp2-dp12, where dp = degree of polymerisation [46], were from Iduron (Manchester, U.K.). Polymeric sulfated heparin derivatives (Table 1) were synthesised in-house as previously described [47]. *N*-sulfated, fluorescein-tagged hexasaccharide glycan substrates (GlcNS–GlcA–GlcNS–IdoA–GlcNS–GlcA-fluorescein, where S = sulfation) containing either l-IdoA or GlcA residues at the third residue from the reducing end (to which a linker and the fluorophore were conjugated) were both purchased from GLYCAN therapeutics (Chapel Hill, NC). All standard laboratory biochemicals were purchased from either Melford or Sigma and were of the highest analytical quality. PAPS (adenosine 3′-phosphate 5′-phosphosulfate, lithium salt hydrate), APS (adenosine 5′-phosphosulfate, sodium salt), PAP (adenosine 3′–5′-diphosphate, disodium salt), CoA (coenzyme A, sodium salt) dephosphoCoA (3′-dephosphoCoA, sodium salt hydrate), ATP (adenosine 5′-triphosphate, disodium salt hydrate), ADP (adenosine 5′-diphosphate, disodium salt), AMP (adenosine 5′-monophosphate, sodium salt), GTP (guanosine 5′-triphosphate, sodium salt hydrate) or cAMP (adenosine 3′,5′-cyclic monophosphate, sodium salt) were all purchased from Sigma and stored at −80°C to minimise degradation. Rottlerin, suramin, aurintricarboxylic acid and all named kinase inhibitors were purchased from Sigma, BD Laboratories, Selleck or Tocris.

**Table 1:** Predominant substitution patterns of differentially sulfated heparin derivatives described in the present study

Analogue	Predominant repeat	IdoUA-2	GlcN-6	GlcN-2	IdoUA-3	GlcN-3a
1 (Heparin)	I_2S_A^6S^Ns	$SO_{3}^{-}$	$SO_{3}^{-}$	$SO_{3}^{-}$	OH	OH
2	I_2S_A^6S^NAc	$SO_{3}^{-}$	$SO_{3}^{-}$	COCH_3_	OH	OH
3	I_2OH_A^6S^Ns	OH	$SO_{3}^{-}$	$SO_{3}^{-}$	OH	OH
4	I_2S_A^6OH^Ns	$SO_{3}^{-}$	OH	$SO_{3}^{-}$	OH	OH
5	I_2OH_A^6S^NAc	OH	$SO_{3}^{-}$	COCH_3_	OH	OH
6	I_2S_A^6OH^NAc	$SO_{3}^{-}$	OH	COCH_3_	OH	OH
7	I_2OH_A^6OH^Ns	OH	OH	$SO_{3}^{-}$	OH	OH
8	I_2OH_A^6OH^NAc	OH	OH	COCH_3_	OH	OH
9	I_2S,3S_A^6S^_3S_Ns	$SO_{3}^{-}$	$SO_{3}^{-}$	$SO_{3}^{-}$	$SO_{3}^{-}$	$SO_{3}^{-}$

#### Cloning, recombinant protein production and SDS–PAGE

Chicken HS2ST (isoform 1), which exhibits ∼92% identity with human HS2ST, was a kind gift from Dr Lars Pedersen (NIH, U.S.A.) and was expressed in the Rosetta-gami (DE3) strain of *Escherichia coli* from a modified pMAL-c2x plasmid encoding an N-terminal maltose-binding protein (MBP) affinity tag. Trimeric recombinant HS2ST1 enzyme was partially purified using immobilised amylose affinity chromatography directly from the cleared bacterial extract, essentially as described previously [28]. MBP-HS2ST was eluted with maltose and further purified by SEC using a HiLoad 16/600 Superdex 200 column (GE Healthcare), which was equilibrated in 50 mM Tris–Cl, pH 7.4, 100 mM NaCl, 10% (v/v) glycerol and 1 mM DTT. Prior to analysis, purified proteins were snap frozen in liquid nitrogen and stored at −80°C. This procedure generated HS2ST of >95% purity. Proteolytic removal of the MBP affinity tag from HS2ST (after re-cloning with MBP and 3C protease sites into the plasmid pOPINM) led to rapid HS2ST denaturation, based on rapid precipitation, so for the procedures described in the present paper the MBP affinity tag was left intact. For SDS–PAGE, proteins were denatured in Laemmli sample buffer, heated at 95°C for 5 min and then analysed by SDS–PAGE with 10% (v/v) polyacrylamide gels. Gels were stained and destained using a standard Coomassie Brilliant Blue protocol. To generate catalytically inactive MBP-HS2ST, the conserved catalytic His residue (His 142) was mutated to Ala using standard PCR procedures [48]. The mutant enzyme was purified as described above.

#### DSF-based fluorescent assays

Thermal shift/stability assays (TSAs) were performed using a StepOnePlus Real-Time PCR machine (Life Technologies) using SYPRO-Orange dye (emission maximum 570 nm, Invitrogen), with thermal ramping between 20 and 95°C in 0.3°C step intervals per data point to induce denaturation in the presence or absence of test biochemicals or small-molecule inhibitors, as previously described [48]. HS2ST was assayed at a final concentration of 5 μM in 50 mM Tris–Cl (pH 7.4) and 100 mM NaCl. Final DMSO concentration in the presence or absence of the indicated concentrations of ligand was no higher than 4% (v/v). Normalised data were processed using the Boltzmann equation to generate sigmoidal denaturation curves, and average *T*_m_/Δ*T*_m_ values were calculated as described using the GraphPad Prism software [48].

#### Microfluidics-based sulfation assay

*N*-sulfated, fluorescein-tagged hexasaccharide glycan substrate (GlcNS–GlcA–GlcNS–IdoA–GlcNS–GlcA-fluorescein, where S = sulfation) containing either l-IdoA or d-GlcA residues at the third residue from the reducing end (to which a linker and the fluorophore were conjugated) was both purchased from GLYCAN therapeutics (www.glycantherapeutics.com). The fluorescein group attached to the reducing end of the glycan substrate possesses a maximal emission absorbance of ∼525 nm, which can be detected by the EZ Reader via LED-induced fluorescence. Chemically modified heparins were generated through a published procedure [47], whereas oligosaccharides from Iduron were generated enzymatically [4,49]. Non-radioactive microfluidic mobility shift carbohydrate sulfation assays were optimised in solution with a 12-sipper chip coated with CR8 reagent and a PerkinElmer EZ Reader II system [50] using EDTA-based separation buffer and real-time kinetic evaluation of substrate sulfation. Pressure and voltage settings were adjusted manually to afford optimal separation of the sulfated product and non-sulfated hexasaccharide substrate, with a sample (sip) volume of 20 nl, and total assay times appropriate for the experiment. Individual sulfation assays were assembled in a 384-well plate in a volume of 80 μl in the presence of the indicated concentration of PAPS or various test compounds, 50 mM HEPES (pH 7.4), 0.015% (v/v) Brij-35 and 5 mM MgCl_2_ (unless specified otherwise). The degree of oligosaccharide sulfation was directly calculated using the EZ Reader software by measuring the sulfo oligosaccharide : oligosaccharide ratio at each time-point. The activity of HS2ST enzymes in the presence of biochemicals and small-molecule inhibitors was quantified in ‘kinetic mode’ by monitoring the amount of sulfated glycan generated over the assay time, relative to control assay with no additional inhibitor molecule (DMSO control). Data were normalised with respect to these control assays, with sulfate incorporation into the substrate limited to ∼20% to prevent depletion of PAPS and substrate and to ensure assay linearity. *K*_m_ and IC_50_ values were determined by nonlinear regression analysis with GraphPad Prism software.

#### NMR-based oligosaccharide sulfation analysis

For NMR experiments, fluorescein-labelled hexasaccharide l-IdoA substrate and the HS2ST-catalysed sulfation product (10 µM) dissolved in 50 mM HEPES, pH 7.4, 5 mM MgCl_2_ and 0.002% (v/v) Brij-35 were lyophilised overnight and re-dissolved in an equivalent amount of D_2_O. NMR experiments were performed at 25°C on a Bruker Avance III 800 MHz spectrometers equipped with a TCI CryoProbe. 1D and 2D proton and TOCSY spectra (mixing time 80 ms) were measured using standard pulse sequences provided by the manufacturer. Spectra were processed and analysed using TopSpin 3.4 software (Bruker).

#### HPLC-based oligosaccharide sulfation analysis

The fluorescein-labelled hexasaccharide l-IdoA substrate and the HS2ST-catalysed sulfation product (10 µM) were analysed after anion-exchange chromatography by HPLC as previously described [51]. Oligosaccharides were digested in the presence of a mixture of heparitinase I, II and III. Samples were loaded on a Proteomix SAX-NP5 (SEPAX) column and eluted with an NaCl gradient. Column effluent was mixed (1 : 1) with 2% (v/v) 2-cyanoacetamide in 250 mM of NaOH and subsequently monitored with a fluorescence detector (JASCO; FP-1520) either at 346 nm excitation and 410 nm emission (detection of mono and disaccharides linked to cyanoacetamide) or at 490 nm excitation and 525 nm emission (for detection of trisaccharides linked to fluorescein).

#### Small-molecule screening assays

The PKIS chemical library (Supplementary Figure S6, designated as SB, GSK or GW compound sets) comprises 367 largely ATP-competitive kinase inhibitors, covering 31 chemotypes originally designed to inhibit 24 distinct protein kinase targets [52]. Compounds were stored frozen as a 10 mM stock in DMSO. The library is characterised as highly drug-like (∼70% with molecular mass <500 Da and clog*P* values <5). For initial screening, compounds dissolved in DMSO were pre-incubated with HS2ST for 10 min and then employed for DSF or sulfotransferase-based enzyme reactions, which were initiated by the addition of the universal sulfate donor PAPS. For inhibition assays, competition assays or individual IC_50_ value determination, a compound range was prepared by serial dilution in DMSO and added directly into the assay to the appropriate final concentration. All control experiments contained 4% (v/v) DMSO, which had essentially no effect on HS2ST activity. Individual chemicals and glycan derivatives were prepared and evaluated using NMR, HPLC, DSF or microfluidics-based assay protocols, as described above.

#### Docking studies

Docking models for rottlerin, suramin and GW407323A were built using Spartan16 (https://www.wavefun.com) and energy minimised using the Merck molecular forcefield. GOLD 5.2 (CCDC Software) was used to dock molecules [53], with the binding site defined as 10 Å around the 5′ phosphorous atom of PAP, using co-ordinates from chicken MBP-HS2ST PDB ID: 4NDZ [20]. A generic algorithm with ChemPLP as the fitness function [54] was used to generate 10 binding modes per ligand in HS2ST. Protons were added to the protein. Default settings were retained for the ‘ligand flexibility’ and ‘fitness and search options’; however, ‘GA settings’ were changed to 200%.

## Results

### Analysis of human HS2ST ligand binding using a TSA

**Figure 1.**
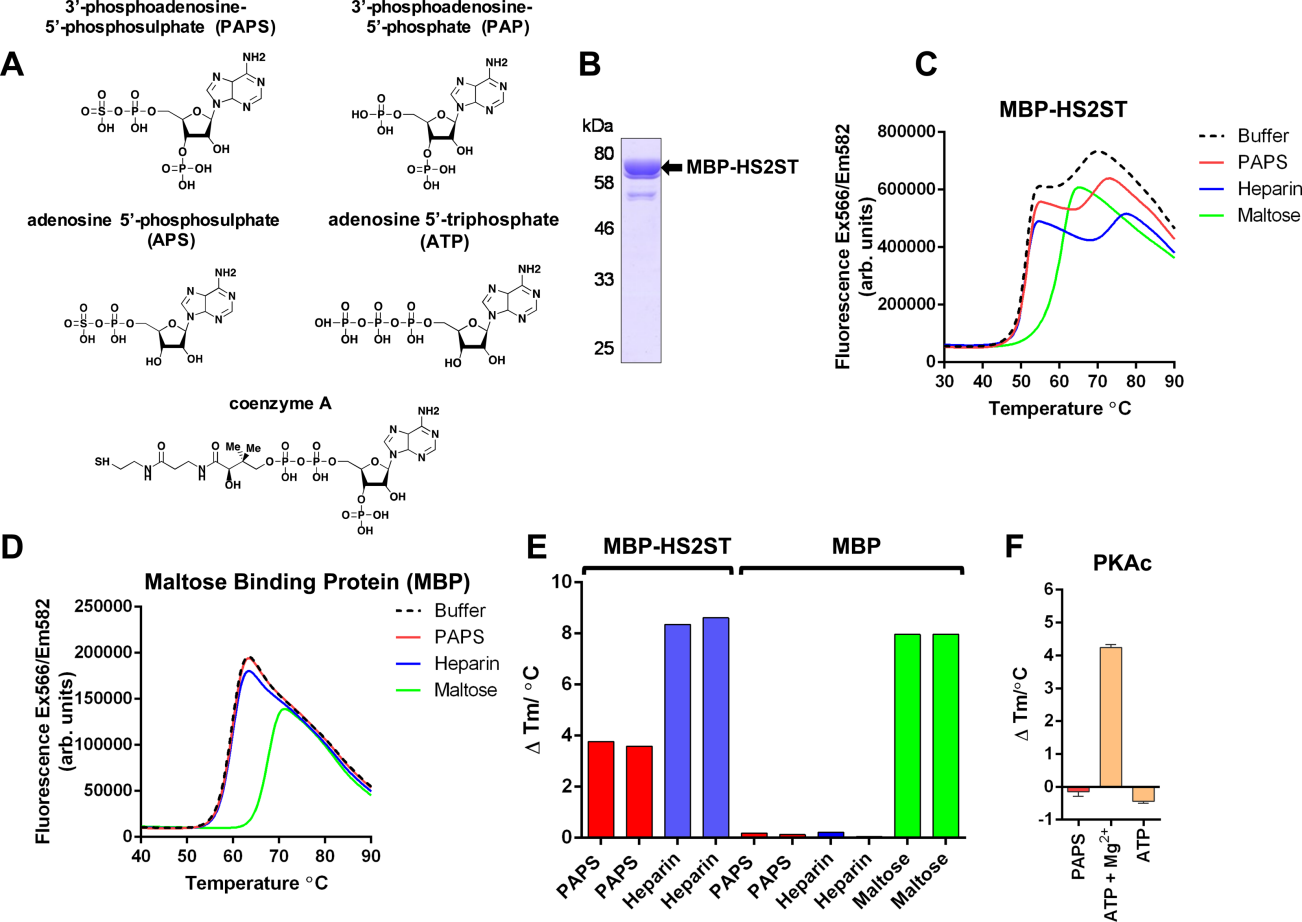
Analysis of purified recombinant MBP-HS2ST protein. (**A**) Structures of PAPS and PAPS-related biochemicals. (**B**) Coomassie blue staining of recombinant MBP-HS2ST1 protein. Approximately 2 μg of purified enzyme was analysed after SDS–PAGE. (**C**) Thermal denaturation profiles of MBP-HS2ST (5 μM) and thermal shift in the presence of 0.5 mM PAPS (red), 10 μM heparin (blue) or 5 mM maltose (green). Buffer control is shown in black dashed lines. (**D**) Thermal denaturation profile of purified recombinant MBP. Experimental conditions as for (**C**). (**E**) *T*_m_ values measured for 5 μM MBP-HS2ST fusion protein (red, blue) or MBP (red, blue, green) in the presence of 0.5 mM PAPS, 10 μM heparin or 5 mM maltose. Δ*T*_m_ values were obtained by DSF and calculated by subtracting control *T*_m_ values (buffer, no ligand) from the measured *T*_m_. (**F**) Δ*T*_m_ values relative to buffer addition for recombinant PKAc (5 μM) measured in the presence of 0.5 mM PAPS, 0.5 mM ATP or 0.5 mM ATP and 10 mM MgCl_2_. Similar results were seen in three independent experiments.

To our knowledge, DSF has not previously been used to examine the thermal stability and thermal shift profiles of sulfotransferases in the presence or absence of biochemical ligands, such as those related to the sulfate donor PAPS (Figure 1A). We purified a recombinant HS2ST catalytic domain (amino acids 69–356) fused to an N-terminal maltose-binding protein (MBP) tag to near homogeneity (Figure 1B) and evaluated its thermal denaturation profile with the MBP tag still attached in the presence of PAPS, heparin or maltose (Figure 1C). As a control, we examined the profile of MBP incubated with the same chemicals (Figure 1D). Unfolding of MBP-HS2ST in buffer generated a biphasic profile, and the upper region of this profile could be positively shifted (stabilised) by incubation with the HS2ST cofactor PAPS or the known HS2ST-interacting oligosaccharide ligand heparin (Figure 1C). In contrast, maltose incubation with MBP-HS2ST induced the same characteristic stabilisation profile observed when MBP was incubated with maltose and then analysed by DSF (Figure 1D). As expected, neither PAPS nor heparin induced stabilisation of MBP, confirming that effects on MBP-HS2ST were due to interaction with the sulfotransferase domain, rather than the affinity tag of the recombinant protein (Figure 1D, relevant Δ*T*_m_ values presented in Figure 1E). Consistently, PAPS did not stabilise the catalytic domain of the ATP-dependent catalytic subunit of cAMP-dependent protein kinase (PKAc), which instead binds with high affinity to the cofactor Mg-ATP [48], inducing a Δ*T*_m_ of >4°C (Figure 1F).

We next analysed the sensitivity of this assay for measuring HS2ST stability shifts over a wide range of PAPS concentrations, which confirmed dose-dependent stabilisation of recombinant HS2ST by PAPS, with detection of binding in the low micromolar range of the cofactor, equivalent to a molar ratio of ∼1 : 1 HS2ST : PAPS (Supplementary Figure S1A). Subsequently, we explored the potential of this assay to detect binding of a putative IdoA-containing oligosaccharide substrate for HS2ST, confirming dose-dependent effects of this polymeric glycan over a range of concentrations, consistent with binding and conformational stability. Similar to PAPS, detection of binding was observed in the low micromolar range, equivalent to a molar ratio of ∼1 : 1 HS2ST : glycan (Supplementary Figure S1B). We also evaluated binding of a panel of adenine-based cofactors (PAP and ATP), which suggested the binding of divalent cation Mg^2+^ ions in an EDTA-sensitive manner (Supplementary Figure S1C), inducing a Δ*Τ*_m_ of ∼3°C, similar to that observed with the HS2ST cofactor PAPS. In contrast, removal of the sulfo moiety of PAPS, which creates the enzymatic end-product PAP, did not abrogate HS2ST binding (Supplementary Figure S2A), consistent with structural analysis of the enzyme [28]. Neither PAP nor PAPS binding required Mg^2+^ ions, although the effect on stabilisation with Mg^2+^ ions was additive (Supplementary Figures 1C and 2A). The non-functional enzyme cofactor APS, in which the 3′-phosphate group of adenine is absent, did not induce HS2ST stabilisation, confirming a requirement for this charged modification (Supplementary Figure S2A). We also established that CoA and acetyl CoA, which both contain a 3′-phosphoadenine moiety, clearly induced thermal stabilisation of HS2ST; loss of the 3′-phosphate group in dephospho-CoA abolished this effect (Supplementary Figure S2A). Finally, we demonstrated that ATP, GTP and ADP, but not AMP or cAMP, were all effective at protecting HS2ST from thermal denaturation, suggesting that they are also HS2ST ligands (Supplementary Figure S2A).

### Analysis of human HS2ST glycan binding using TSA

To extend our HS2ST thermal analysis to identify potential glycan substrates, we evaluated enzyme stability in the presence of synthetic glycan chains of different lengths and sulfation patterns (Table 1). Of particular interest for further assay development, thermal shift (stabilisation) was detected in this assay when hexasaccharide (dp6) or a higher degree of polymerisation oligosaccharide was incubated with the enzyme (Supplementary Figure S2B), suggesting that a dp6 glycan might represent the shortest potential partner suitable for HS2ST binding, a prerequisite for enzymatic modification. Interestingly, many of the chemically modified heparins tested served as efficient HS2ST-binding partners relative to the heparin control. The fully chemically sulfated I_2s,3s_A^6s^_3s_Ns hexamer induced a similar HS2ST stability shift to heparin, whereas the singly and doubly desulfated hexamers induced slightly smaller stability shifts (Supplementary Figure S2C). Moreover, a putative I_2OH_A^6OH^Ns substrate, which contains a 2-O moiety that is predicted to be the substrate for 2-*O*-sulfotransferases, also led to marked thermal stabilisation of HS2ST, suggestive of productive binding to HS2ST that might permit it to be sulfated in the presence of PAPS (Supplementary Figure S2C).

### A novel microfluidic kinetic assay to directly measure oligosaccharide sulfation by HS2ST

**Figure 2.**
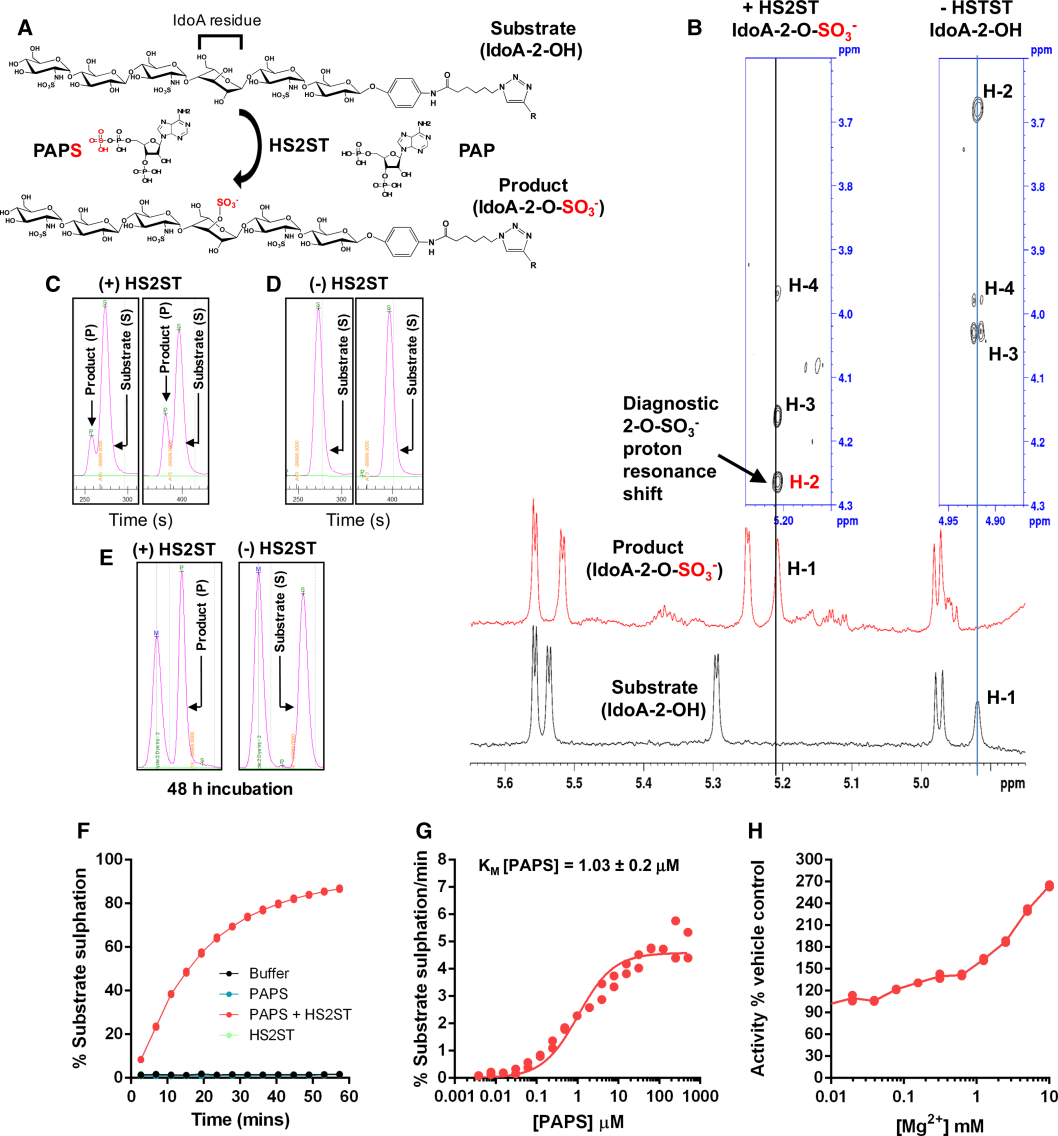
Development of a novel microfluidic mobility shift assay to quantify HS2ST enzymatic activity. (**A**) Schematic showing PAPS-dependent sulfate incorporation into the fluorescein-labelled hexasaccharide IdoA substrate by HS2ST, with the concomitant generation of PAP. R = fluorescein. (**B**) NMR analysis of the non-sulfated and sulfated hexasaccharides. The addition of a 2-*O*-sulfate group to the iduronate (l-IdoA) residue of the fluorescent hexasaccharide results in a significant chemical shift change, most notably to the anomeric proton (H-1) and that of H-2 attached to the sulfated carbon atom of l-IdoA, in agreement with expected values from the literature [47]. ^1^H NMR spectrum of non-sulfated substrate (bottom spectrum, black) and sulfated product (upper spectrum, red). Distinct l-IdoA protons (H-3 and H-4 of the spin system) were identified by TOCSY and are shown vertically above their respective H-1 signals (for the non-sulfated substrate, right blue boxed, and for the sulfated product, left blue boxed). The full carbohydrate proton spectra are shown in Supplementary Figure S3. (**C** and **D**) Screen shots of EZ reader II raw data files, demonstrating that HS2ST induces a rapid mobility change in the IdoA-containing fluorescent hexasaccharide. Separation of the higher mobility, sulfated (product, P) from the lower mobility (substrate, S) hexasaccharide occurs as a result of enzymatic substrate sulfation (left panels 180 s assay time, right panels 240 s assay time), as demonstrated by omission of HS2ST from the assay (−HS2ST). Assays were initially performed at 20°C using 90 nM of purified HS2ST, 2 μM fluorescein-labelled hexasaccharide substrate and 500 μM PAPS. (**E**) Stoichiometric sulfate-labelling of IdoA-containing fluorescein-labelled hexasaccharide. Reactions were performed with 0.6 μM HS2ST, 375 μM IdoA-hexasaccharide substrate and 1 mM PAPS and incubated at room temperature for 48 h. The reaction was spiked with an additional 0.5 mM (final concentration) of PAPS after 24 h of incubation. M = non-sulfated marker substrate. A final hexasaccharide concentration of 2 μM was analysed by the fluorescent sulfation mobility assay. (**F**) Analysis of time-dependent sulfate incorporation into 2 μM IdoA-containing fluorescein-conjugated hexasaccharide. Percentage sulfation was calculated from the ratio of substrate hexasaccharide to product (2-*O*-sulfo)-hexasaccharide at the indicated time points in the presence or absence of 20 nM HS2ST and 10 μM PAPS. (**G**) Calculation of *K*_m_ [PAPS] value for HS2ST. PAPS concentration was varied in the presence of a fixed concentration of HS2ST (20 nM), and the degree of substrate sulfation calculated from a differential kinetic analysis, *n* = 2 assayed in duplicate. (**H**) Duplicate HS2ST assays conducted in the presence of increasing concentrations of activating Mg^2+^ ions. Activity is presented in duplicate relative to buffer controls. Similar results were seen in several independent experiments.

To quantify the effects of various ligands on HS2ST enzyme activity, we sought to develop a new type of rapid non-radioactive solution assay that could discriminate the enzymatic incorporation of sulfate into a synthetic oligosaccharide substrate. Current protocols are time-consuming and cumbersome, requiring mass spectrometry, NMR or ^35^S-based radiolabelling/HPLC separation procedures. Importantly, we next tested whether a version of an I_2OH_A^6OH^NS containing a hexasaccharide substrate coupled to a linker and fluorescein at the reducing end, which interacts with HS2ST (Supplementary Figure S2C), could also be enzymatically sulfated by HS2ST using ‘gold-standard’ NMR-based sulfation detection [47]. The fluorescent I_2OH_A^6OH^Ns could not be evaluated for binding to HS2ST by DSF, due to interference of the fluorescent group in the unfolding assay, which measures SYPRO-Orange fluorescence at a similar wavelength. Instead, to confirm sulfation of the fluorescein-labelled substrate, it was pre-incubated with PAPS and HS2ST to catalyse site-specific sulfation (Figure 2A). The NMR spectrum of the sulfated product compared with that of the non-modified substrate provided unequivocal evidence for sulfation at the 2-O position of the sugar, most notably due to the diagnostic shifts of anomeric H-1 and H-2 protons in the presence of the 2-*O*-sulfate group linkage to the carbon atom (Figure 2B and Supplementary Figure S3). The 2-*O-*sulfated IdoA hexameric product was also confirmed using an established HPLC-based approach [51], which demonstrated stoichiometric sulfation of an enzyme-derived substrate derivative (Supplementary Figure S4).

Next, we evaluated the incorporation of the sulfate moiety from PAPS into a fluorescently labelled glycan substrate using a microfluidic assay that detects real-time changes in substrate covalent modification (notably the introduction of a negative charge) when an electric field is applied to the solution reaction. This ratiometric assay, which we and others have previously employed to detect the formal double-negative charge induced by real-time peptide phosphorylation [43,55–57], was able to detect real-time incorporation of sulfate into the oligosaccharide substrate, based on the different retention time of the product compared with the substrate (Figure 2C). No sulfated product was detected in the absence of HS2ST (Figure 2D), and prolonged incubation of substrate with HS2ST led to stoichiometric conversion of the substrate into the fully sulfated product (P), which migrated very differently to the substrate (S) ‘marker’ (Figure 2E). Analysis of product/(product + substrate) ratios of the peak heights allowed us to monitor sulfation over any appropriate assay time (Figures 2F), and the degree of sulfation could easily be varied as a function of PAPS concentration in the assay. Furthermore, no sulfated product was detected in the presence of buffer or PAPS alone (Figure 2F), allowing us to determine a *K*_m_ value of ∼1 μM for PAPS-mediated substrate hexasaccharide sulfation (Figure 2G). We also noted that high (>1 mM) concentrations of Mg^2+^ ions led to concentration-dependent increases in enzyme HS2ST activity (Figure 2H), consistent with the effects of Mg^2+^ ions identified in DSF assays (Supplementary Figure S2A). Next, we confirmed that sulfation was optimal when an appropriate modifiable IdoA substrate was present, with sulfation reduced by >90% when a GlcA residue was incorporated into the central disaccharide of the substrate instead (compare Supplementary Figure 5A,B). To further validate our assay, we evaluated a catalytically inactive point mutant of HS2ST, in which the putative catalytic base (His142) was mutated to Ala [28]. Purified H142A MBP-HS2ST appeared to be appropriately folded, and although it bound to PAPS and heparin (Supplementary Figure S6A–C), it was unable to efficiently catalyse sulfation of the fluorescent I_2OH_A^6OH^NS hexasaccharide substrate, possessing <1% of the activity observed with wild-type MBP-HS2ST (Supplementary Figure S6D).

### Screening for small-molecule inhibitors of HS2ST using DSF and microfluidic technology

**Figure 3.**
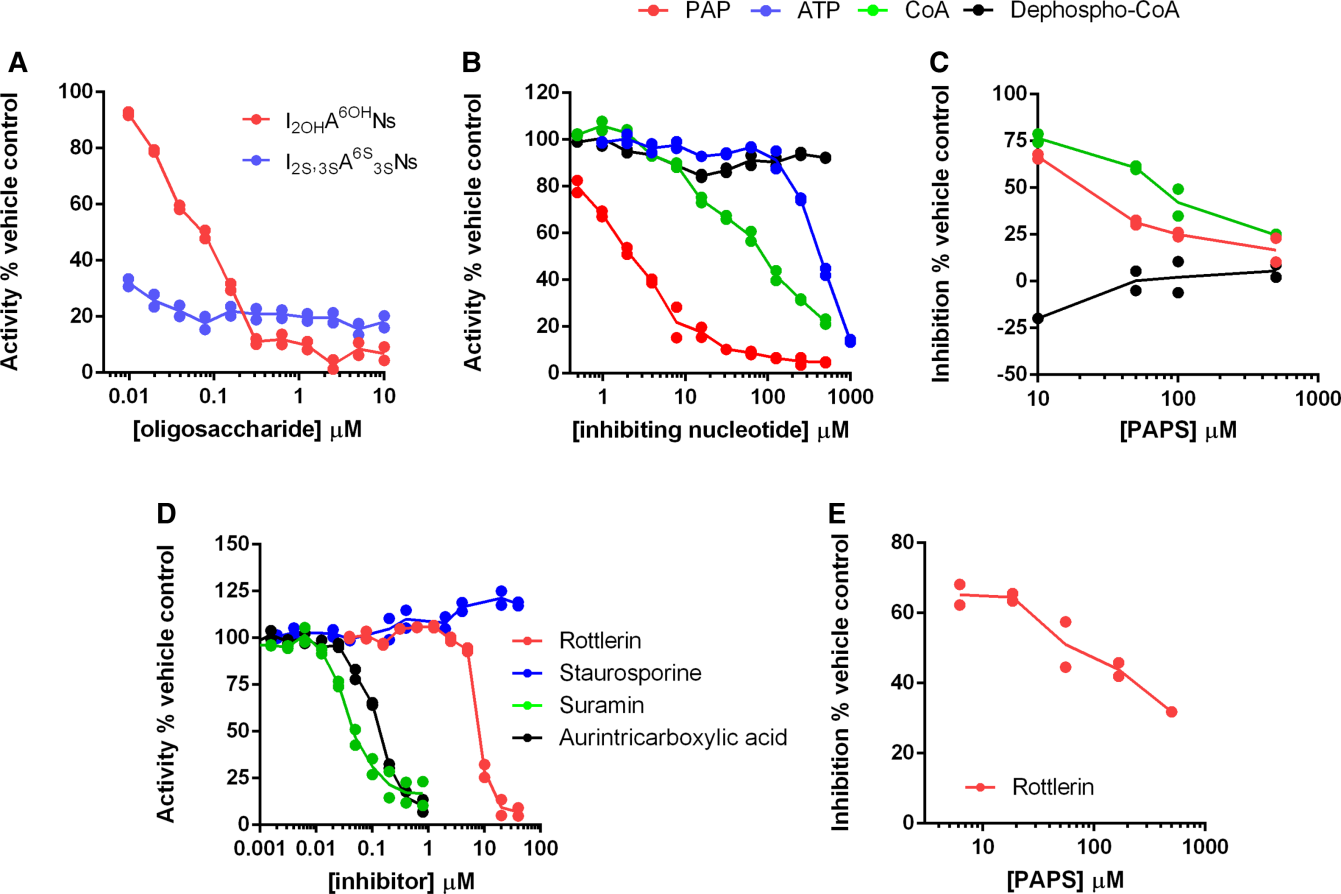
Microfluidic sulfotransferase assay to measure inhibition of HS2ST activity *in vitro*. Assays were performed using 20 nM HS2ST and the extent of substrate sulfation was determined after 15 min incubation at room temperature, as described in Figure 2. Dose–response curves for inhibition of HS2ST activity by (**A**) modified heparin derivatives containing different sulfation patterns (assayed in the presence of 0.5 mM MgCl_2_) or (**B**) nucleotides (assayed in the absence of MgCl_2_). Assays contained HS2ST and 10 μM PAPS and the indicated concentration of inhibitory ligand or buffer. (**C**) Inhibition of HS2ST activity by fixed 10 μM PAP, 0.5 mM CoA or 0.5 mM dephospho-CoA in the presence of increasing concentration of PAPS. Inhibition is calculated as a function of no inhibitor for each concentration of PAPS in the absence of MgCl_2_. (**D**) Evaluation of small-molecule HS2ST inhibitory profiles in the presence of 10 μM PAPS. (**E**) Inhibition of HS2ST activity by 20 μM rottlerin in the presence of varied concentrations of PAPS, suggesting a competitive mode of inhibition. Similar results were seen in multiple experiments.

The discovery of HS2ST inhibitors is hindered by a lack of a rapid and quantifiable assay for the facile detection of sulfate modification using a close mimic of a physiological substrate. Our discovery that a synthetic HS2ST glycan substrate could be readily sulfated and detected by enzymatic assay in solution, without the need for HPLC, NMR or radioactive procedures, meant that this approach might now be optimised for the discovery of small-molecule HS2ST inhibitors. We first evaluated the ability of an unlabelled (non-fluorescent) heparin glycan substrate that lacked sulfate at the 2-O position, or a non-substrate heparin that was fully sulfated at all potential sites, to act as HS2ST inhibitors in our fluorescent glycan sulfation assay. As detailed in Figure 3A, the fully sulfated glycan was a potent inhibitor, interfering with HS2ST-dependent sulfation of the substrate with an IC_50_ value of <10 nM, consistent with tight binding to the enzyme, as previously established using DSF (Supplementary Figure S2C). In contrast, a less highly sulfated substrate was still able to compete with the fluorescent substrate in a dose-dependent manner (fixed at 2 μM in this assay), as indicated by the IC_50_ value of <100 nM. We next compared the effects of PAP, ATP, CoA and dephospho-CoA, which all exhibit thermal stabilisation of HS2ST in DSF assays (Supplementary Figure S2A). Interestingly, PAP (IC_50_ ∼2 μM), CoA (IC_50_ = 65 μM) and ATP (IC_50_ = 466 μM) were HS2ST inhibitors, whereas dephospho-CoA (which lacks the 3′-phosphate moiety in CoA) was not (Figure 3B). Increasing the concentration of PAPS in the assay led to a decrease in the level of inhibition by both PAP and CoA (Figure 3C), suggesting a PAPS-competitive mode of inhibition, as predicted from the various shared chemical features of these molecules (Figure 1A).

Recent studies have demonstrated that PAPS-dependent tyrosyltransferases (tyrosyl protein sulfotranferase, TPSTs) are inhibited by non-nucleotide-based polyanionic chemicals [58]. However, to our knowledge, the inhibition of carbohydrate sulfotransferases by such compounds has not been reported. Using our microfluidic assay, we confirmed that the polysulfated compound suramin (an inhibitor of angiogenesis) and the polyaromatic polyanion aurintricarboxylate (an inhibitor of protein : nucleic acid interactions, DNA polymerase and topoisomerase II) demonstrated nanomolar inhibition of HS2ST, with IC_50_ values of 40 ± 1 and 123 ± 7 nM, respectively (Figure 3D). In addition, the non-specific protein kinase inhibitor rottlerin also inhibited HS2ST with an IC_50_ of 6.4 μM. Increasing the concentration of PAPS in the sulfation assay decreased the inhibitory effect, consistent with a competitive mode of HS2ST inhibition for rottlerin (Figure 3E).

### Protein kinase inhibitors are a new class of potential broad-spectrum HS2ST inhibitor

**Figure 4.**
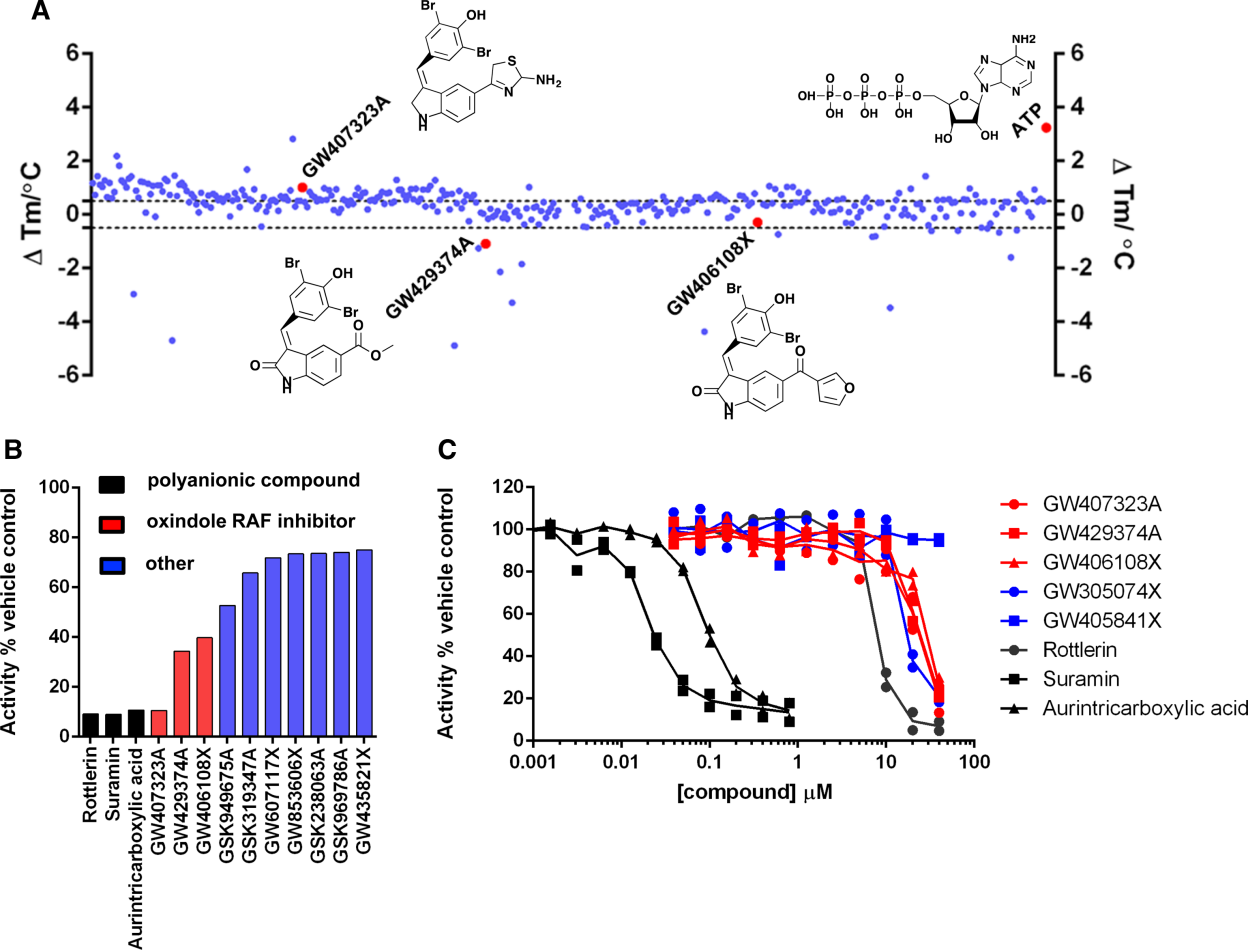
Mining the PKIS inhibitor library for HS2ST inhibitor compounds. (**A**) Evaluation of small-molecule ligands in a high-throughput HS2ST DSF assay. HS2ST (5 μM) was screened in the presence or absence of 40 μM compound. The final concentration of DMSO in the assay was 4% (v/v). Δ*T*_m_ values (positive and negative) were calculated by subtracting the control *T*_m_ value (DMSO alone) from the measured *T*_m_ value. Data shown on a scatter plot of the mean Δ*T*_m_ values from two independent DSF assays. (**B**) Enzymatic analysis of HS2ST inhibition by selected PKIS compounds. HS2ST (20 nM) was incubated with the indicated PKIS compound (40 μM) in the presence of 10 μM PAPS for 15 min at room temperature. HS2ST sulfotransferase activity was assayed using the fluorescent hexasaccharide substrate and normalised to DMSO control (4%, v/v). (**C**) Full dose–response curves for selected compounds. HS2ST (20 nM) was incubated with increasing concentration of inhibitor in the presence of 1 μM PAPS for 15 min at 20°C. HS2ST activity calculated as above. Data from two independent experiments are combined. Similar results were seen in an independent experiment.

The finding that the non-specific kinase inhibitor rottlerin [59] was a micromolar inhibitor of HS2ST was of particular interest, especially given the remarkable progress in the development of kinase inhibitors as chemical probes, tool compounds and, latterly, clinically approved drugs. Similarities between ATP and PAPS (Figure 1A), and the finding that ATP can both bind to, and inhibit, HS2ST activity (Supplementary Figure S2A and Figure 3B) raised the possibility that other ATP-competitive protein kinase inhibitors might also interact with HS2ST. To exploit our screening capabilities further, we established a 384-well assay to evaluate inhibition of PAPS-dependent glycan sulfation by HS2ST. The Published Kinase Inhibitor Set (PKIS) is a well-annotated collection of 367 high-quality ATP-competitive kinase inhibitor compounds that are ideal for compound repurposing or the discovery of new chemical ligands for orphan targets. We screened PKIS using DSF and enzyme-based readouts (Figure 4A,B, respectively). As shown in Figure 4A, when screened at 40 μM compound in the presence of 5 μM HS2ST, only a small percentage of compounds induced HS2ST stabilisation or destabilisation at levels similar to that seen with an ATP control. We focussed on compounds inducing HS2ST Δ*T*_m_ values between +0.5°C and −0.5°C, and re-screened each ‘hit’ compound using ratiometric HS2ST enzyme assays at a final compound concentration of 40 μM. We reported the enzyme activity remaining compared with DMSO, with rottlerin (IC_50 _= ∼8 μM), suramin (IC_50 _= ∼20 nM) and aurintricarboxylate (IC_50_ = ∼90 nM) as positive controls (Figure 4B and Supplementary Figures 7 and 8). We also included the compound GW406108X in our enzyme assay since it was structurally related to several ‘hit’ compounds from the DSF screen. As shown in Figure 4C, the three PKIS compounds with the highest inhibitory activity (red) exhibited IC_50_ values of between 20 and 30 μM towards HS2ST in the presence of 1 μM PAPS, similar to inhibition by rottlerin. Of particular interest, these three compounds were among the top ∼10% of compounds in terms of their Δ*T*_m_ values (red spheres, Figure 4A). Chemical deconvolution of compounds revealed that all three were closely related members of a class of oxindole-based RAF protein kinase inhibitor (Figure 4A). Subsequently, one other related indole RAF inhibitory compound from PKIS, GW305074, was also shown to be a mid-micromolar HS2ST inhibitor, whereas the related oxindole GW405841X (Supplementary Figure S8) did not inhibit HS2ST at any concentration tested (Figure 4C). Finally, we used combined DSF and enzyme assays to evaluate a broader panel of well-characterised kinase inhibitors (Supplementary Figure S9). Interestingly, neither the pan-kinase inhibitor staurosporine nor several FDA-approved tyrosine kinase inhibitors caused thermal stabilisation of HS2ST at any concentration tested. Moreover, chemically diverse RAF inhibitors, including clinical RAF compounds such as dabrafenib and vemurafenib, were unable to inhibit HS2ST in our sensitive HS2ST enzyme, even at concentrations as high as 400 μM (Supplementary Figure S9B).

### Docking analysis of HS2ST ligands

**Figure 5.**
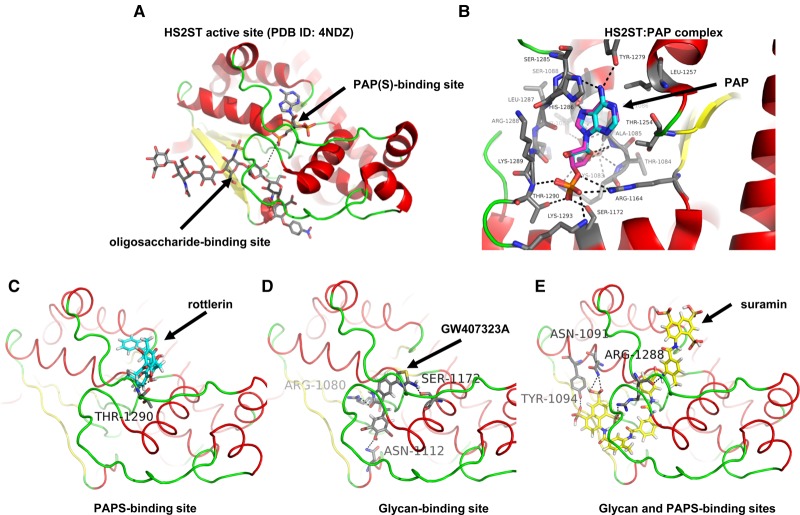
Molecular docking analysis of HS2ST with small-molecule inhibitor compounds. (**A**) Structural representation of the catalytic domain of chicken MBP-HS2ST crystallised with bound heptasaccharide and non-sulfated PAP cofactor (protein rendered as a cartoon). Red — α-helix, yellow — β-sheet, green — loop. PAP (adenosine-3′-5′-diphosphate) and heptasaccharride are rendered as coloured sticks. Grey — carbon, red — oxygen, blue — nitrogen, yellow — sulfur. Black dotted line indicates close proximity of glycan 2-OH group and PAP. (**B**) Structure of HS2ST with near-identical crystallographic (carbons in cyan) and docking (carbons in purple) poses of PAP (protein rendered as a cartoon). Red — α-helix, yellow – β-sheet, green — loop. PAP rendered as coloured sticks. Cyan/grey/purple — carbon, red — oxygen, blue — nitrogen, dark yellow — sulfur). Black dotted lines indicate hydrogen bonds. Molecular docking of (**C**) rottlerin and (**D**) the indole RAF inhibitor GW407323A or (**E**) suramin into the HS2ST catalytic domain (protein depicted as a cartoon). Red — α-helix, yellow — β-sheet, green — loop. Docked molecules coloured as sticks. Pink/yellow/salmon/grey — carbon, red — oxygen, blue — nitrogen, dark yellow — sulfur, white — hydrogen). Black dotted lines indicate hydrogen bonds. Amino acid numbering corresponds to that of trimeric HS2ST.

The X-ray structure (PDB ID: 4NDZ) of trimeric chicken MBP-HS2ST fusion protein bound to non-sulfated PAP (adenosine-3′-5′-diphosphate, a potent HS2ST inhibitor that was identified in the present study) and a polymeric oligosaccharide have previously been reported [20,28]. We employed a 3.45 Å structural dataset to dock rottlerin, suramin and the most potent oxindole-based ‘hit’ from the screen (GW407323A, see Figure 4B) into the extended enzyme active site. As shown in Figure 5A, HS2ST possesses substrate-binding features that accommodates an extended oligosaccharide that place it in close proximity to the desulfated PAP end-product, which substitutes for the endogenous PAPS cofactor during crystallisation. The 3′-phosphoadenine moiety of PAP also helps anchor the nucleotide in an appropriate position. A molecular docking protocol for PAP in HS2ST was developed that matched the crystallographic binding pose of PAP extremely well (RMSD 0.31 Å, Figure 5B). By comparing a crystallised ligand (PAP) with docked rottlerin, suramin and GW407323A, we confirmed that compounds could be docked into the active site of HS2ST broadly corresponding to either the PAPS-binding region (rottlerin and GW407323A, Figure 5C,D) or bridging both the substrate and cofactor-binding sites (suramin, Figure 5E). In these binding modes, compounds make many stabilising amino acid interactions that permit them to compete with PAPS or oligosaccharide substrate for binding to HS2ST (Figure 5C, residue numbering based on the HS2ST trimer). For example, rottlerin is predicted to form a hydrogen bond with the amide backbone of Thr 1290, GW407323A has multiple potential hydrogen bonding interactions with residues including Arg 1080, Asn 1112 and Ser 1172, while suramin is predicted to form hydrogen bonds with residues Asn 1091, Tyr 1094 and Arg 1288, allowing this highly elongated inhibitor to straddle separate regions of the active site.

## Discussion

In the present paper, we report a simple method for the detection of enzyme-catalysed glycan sulfation using a model IdoA-containing hexasaccharide fused to a reducing-end fluorophore. The chemical similarity between ATP, a universal phosphate donor, and PAPS, a universal sulfate donor, led us to investigate whether enzymatic glycan sulfation could be detected using a high-throughput kinetic procedure previously validated for peptide phosphorylation by ATP-dependent protein kinases. We focussed our attention on HS2ST, which transfers sulfate from PAPS to the 2-O position of IdoA during heparan sulfate biosynthesis in the secretory pathway.

To facilitate rapid purification of recombinant HS2ST, the enzyme was expressed as an N-terminal MBP fusion protein, and we confirmed that it was folded, and could bind to a variety of known exogenous ligands including PAPS and PAP, the end-product of the sulfotransferase reaction. Protein kinases are also known to bind to their end-product (ADP), and kinase structural analysis has long taken advantage of the stability of kinase and ATP analogues, or ADP-like complexes, for protein co-crystallisation. Similar co-crystallisation approaches revealed the structure of HS2ST, and related sulfotransferases, in complex with PAP and model saccharide substrates [20,21], and our study extends these approaches, by revealing a competitive mode of HS2ST interaction with a variety of 3′-phosphoadenosine-containing nucleotides, including CoA. They also suggest that generalised docking of a 3′phosphoadenosine moiety is a feature of HS2ST that could be mimicked using other small-molecule inhibitors. DSF-based thermal shift assays are ideal for the analysis of a variety of proteins and ligands, including growth factors [4,60], protein kinase domains [45,48,57], pseudokinase domains [61,62], BH3 [63] and bromodomain-containing proteins [64]. However, to our knowledge, this is the first report to demonstrate the utility of a DSF-based strategy for the analysis of any sulfotransferase.

### Competitive HS2ST inhibition by biochemical ligands

By developing a new type of rapid, kinetic glycan sulfation assay, we confirmed that many HS2ST ligands also act as competitive inhibitors of PAPS-dependent oligosaccharide sulfation, setting the stage for a broader screening approach for the discovery of HS2ST inhibitors. Standard assays for carbohydrate sulfation utilise HPLC-based detection of ^35^S-based substrate sulfation derived from ^35^S-labelled PAPS, requiring enzymatic cofactor synthesis and time-consuming radioactive solid-phase chromatography procedures [20,35,41]. While enzymatic deconvolution, MS and NMR-based procedures remain useful for mapping sulfation patterns in complex (sometimes unknown) glycan polymers, these procedures are very time-consuming and relatively expensive. In contrast, our finding that sulfation can be detected using a simple glycan mobility shift assay, and then quantified in real time by comparing the ratio of a sulfated and non-sulfated substrate, is rapid, reproducible and relatively inexpensive. Our kinetic assay makes use of a commercial platform originally developed for the analysis of peptide phosphorylation or peptide proteolysis, which allows for the inclusion of high concentrations of non-radioactive cofactors, substrates and ligands in assays [45]. Consequently, we were able to use this technology to derive a *K*_m_ value for PAPS in our standard HS2ST assay of 1.0 μM (Figure 2G), slightly lower than the reported literature value of 18.5 μM for HS2ST using desulfated heparin as a substrate [20], but similar to the reported literature value of ∼4.3 μM for the PAPS-dependent GlcNAc-6-sulfotransferase NodH from *Rhizobium melitoli* [35] and 1.5 and 10 μM for human hormone iodotyrosine sulfotransferases and tissue-purified tyrosyl sulfotransferase [65,66]. In the course of our studies, we developed several new reagents, including a hexameric fluorescent substrate in which the central IdoA residue was replaced by a GlcA residue (Supplementary Figure S5). Interestingly, a decreased rate of substrate modification was observed using this oligosaccharide substrate, consistent with the ability of HS2ST to sulfate either IdoA or GlcA [19], but with a marked preference for the former. Previous HPLC-based studies identified an N-sulfo group in the oligosaccharide substrate as a prerequisite for catalysis, with subsequent preferential transfer of sulfate to the 2-O position of IdoA [20,22,28,67]; these published observations are entirely consistent with our findings using a hexameric fluorescent substrate.

In the future, it might be possible to quantify other site-specific covalent modifications in complex glycans using fluorescent oligosaccharides that contain distinct sugar residues, and by employing mobility-dependent detection in the presence of a variety of enzymes. These could include 3-*O-* and 6-*O-*sulfotransferases [21] or structurally distinct glycan phosphotransferases, such as the protein-*O*-mannose kinase POMK/Sgk196 [68], which catalyses an essential phosphorylation step during biosynthesis of an α-dystroglycan substrate [69]. Using this general approach, the screening and comparative analysis of small-molecule inhibitors of these distinct enzyme classes would be simplified considerably relative to current procedures.

### HS2ST inhibition by known kinase inhibitors, including a family of known RAF inhibitors

Our finding that HS2ST was inhibited at sub-micromolar concentrations by the compounds suramin [70] and the DNA polymerase inhibitor aurintricarboxylic acid [71] was intriguing, and consistent with recent reports demonstrating inhibitory activity of these compounds towards TPSTs, which employ PAPS as a cofactor, but instead sulfate tyrosine residues in specific motifs embedded in a variety of proteins [58]. During the course of our studies screening a panel of kinase inhibitors, we found that the non-specific kinase compound rottlerin is a micromolar inhibitor of HS2ST *in vitro*, with inhibition dependent on the concentration of PAPS in the assay, suggesting a competitive mode of interaction. Rottlerin (also known as mallotoxin) is a polyphenolic compound from *Mallotus philippensis* and, although originally identified as an inhibitor of PKC isozymes [72], possesses a wide variety of biological effects likely due to its non-specific inhibition of multiple protein kinases [59]. This lack of specificity prevents exploitation of rottlerin in cells as a specific probe, although our finding that HS2ST is a target of this compound opens up the possibility that this, or other, protein kinase inhibitors might also possess inhibitory activity towards HS2ST, either due to an ability to target the PAPS or oligosaccharide-binding sites in the enzyme. To evaluate these possibilities further, we screened PKIS, a collection of drug-like molecules with broad inhibitory activity towards multiple protein kinases. Interestingly, only three compounds (<1% of the library) consistently showed marked inhibitory activity at 40 μM in our HS2ST enzyme assay (Figure 4A–C, red). Remarkably, all three compounds belonged to the same benzylidene-1H-inol-2-one (oxindole) chemical class, which were originally reported as potent ATP-dependent RAF kinase inhibitors that block the MAPK signalling pathway in cultured cells [73]. Retrospectively, of all the related chemotypes present in the PKIS library, we confirmed that GW305074X (but not GW405841X) was also a micromolar HS2ST inhibitor, consistent with the broad sensitivity of HS2ST to this optimised class of RAF inhibitor.

Although limited structure–activity relationships can be derived from our initial studies, these findings demonstrate that HS2ST inhibitors can be discovered, and that several of these inhibitors could be of broad interest to the sulfotransferase (and protein kinase) fields. An additional outcome of our work is that pharmaceutical companies might conduct more extensive high-throughput screens using much larger libraries of kinase inhibitors to identify distinct, and more potent, leads. Our study also validates previous observations from the turn of the century, in which carbohydrate inhibitors of NoDH sulfotransferase were reported from a low diversity kinase-directed library [35]. Surprisingly, this early breakthrough did not lead to the development of any glycan sulfotransferase tool compounds for cell-based analysis. However, our discovery that oxindole-based RAF inhibitors are also HS2ST inhibitors could provide new impetus for the design and synthesis of much more specific and potent HS2ST inhibitors from this class of RAF kinase inhibitor, especially if issues of specificity can be evaluated using mutagenic target-validation approaches previously validated for various protein kinases [74–76].

A requirement for rapid progress during this process will be structure-based analysis of HS2ST in the presence of compounds, in order to determine mechanism and mode(s) of interaction. Our initial docking studies suggest similar binding modes for both rottlerin and the oxindole-based ligand GW407323A (Figure 5), with the potential for cross-over between PAPS and substrate-binding sites present on the surface of HS2ST. It will be intriguing to explore these binding modes by structural analysis and guided mutational approaches [77], in order to evaluate potential drug-binding site residues in HS2ST and to tease apart requirements for enzyme inhibition. It will also be important to assess whether compounds identified as *in vitro* HS2ST inhibitors, including previously reported RAF inhibitors, can also interfere with HS sulfation and downstream signalling in cells. Interestingly, suramin is a potent anti-angiogenic compound and is reported to have cellular effects on FGF signalling [78], whereas aurintricarboxylate has multiple cellular effects currently attributed to nucleotide-dependent processes. Attempting to link some of these cellular phenotypes to the inhibition of 2-*O* glycan sulfation is a worthy future experimental strategy, although success with PAPS-competitive compounds is likely to depend on the concentration of PAPS in the Golgi network and the relative rate of, minimally, 2-*O-*sulfate turnover (sulfation versus desulfation) among physiological HS2ST substrates.

## Conclusion

Our work raises the possibility that HS2ST inhibitors could be developed strategically following the successful blueprint laid down for protein kinase inhibitors in the previous decades. Dozens of sulfotransferases are found in vertebrate genomes, and the development of chemical biology approaches to rapidly inactivate Golgi membrane-bound sulfotransferases and induce targeted inhibition of sulfation has been stymied by a lack of tool compounds, whose exploitation has the opportunity to revolutionise cell biology when properly validated [79,80]. We propose that if such compounds can be developed, perhaps through high-throughput screening and discovery of new inhibitors, or even via chemical manipulation of the leads reported in the present study, then a new era in sulfation-based cell biology might be on the horizon. By generating tools to chemically control glycan sulfation modulated by HS2ST directly, inhibitor-based interrogation of sulfation-dependent enzymes could also have significant impact in many active areas of translational research.

## Abbreviations

DSF: differential scanning fluorimetry
GlcA: β-d-glucouronate
HS2ST: heparan sulfate 2-*O*-sulfotransferase
IdoA: α-l-iduronate
MBP: maltose-binding protein
PAPS: adenosine 3′-phosphate 5′-phosphosulfate
PKIS: Published Kinase Inhibitor Set
POMK: protein-*O*-mannose kinase
RAF: rapidly accelerated fibrosarcoma
ST: sulfotransferases
TPST: tyrosyl protein sulfotranferase
TSA: thermostability assay

## References

[1] Leung A.W., Backstrom I. and Bally M.B. (2016) Sulfonation, an underexploited area: from skeletal development to infectious diseases and cancer. Oncotarget 7, 55811–55827 10.18632/oncotarget.10046 27322429 PMC5342455

[2] Bowman K.G. and Bertozzi C.R. (1999) Carbohydrate sulfotransferases: mediators of extracellular communication. Chem. Biol. 6, R9–R22 10.1016/S1074-5521(99)80014-3 9889154

[3] Kreuger J., Spillmann, D, Li, J.P. and Lindahl, U. (2006) Interactions between heparan sulfate and proteins: the concept of specificity. J. Cell Biol. 174, 323–327 10.1083/jcb.200604035 16880267 PMC2064228

[4] Li Y., Sun, C., Yates E.A., Jiang C., Wilkinson M.C and Fernig D.G. (2016) Heparin binding preference and structures in the fibroblast growth factor family parallel their evolutionary diversification. Open Biol. 6 10.1098/rsob.150275 PMC4821243 27030175

[5] Tillo M., Charoy C., Schwarz Q., Maden C.H., Davidson K., Fantin A. et al. (2016) 2- and 6-*O*-sulfated proteoglycans have distinct and complementary roles in cranial axon guidance and motor neuron migration. Development 143, 1907–1913 10.1242/dev.126854 27048738 PMC4920156

[6] Chan W.-K., Price D.J. and Pratt T. (2017) FGF8 morphogen gradients are differentially regulated by heparan sulphotransferases Hs2st and Hs6st1 in the developing brain. Biol. Open 6, 1933–1942 10.1242/bio.028605 29158323 PMC5769653

[7] Clegg J.M., Conway, C.D., Howe, K.M., Price, D.J., Mason, J.O., Turnbull, J.E. et al. (2014) Heparan sulfotransferases Hs6st1 and Hs2st keep Erk in check for mouse corpus callosum development. J. Neurosci. 34, 2389–2401 10.1523/JNEUROSCI.3157-13.2014 24501377 PMC3913879

[8] Chan W.K., Howe K., Clegg J.M., Guimond S.E., Price D.J., Turnbull J.E. et al (2015) 2-*O* heparan sulfate sulfation by Hs2st is required for Erk/Mapk signalling activation at the mid-gestational mouse telencephalic midline. PLoS ONE 10, e0130147 10.1371/journal.pone.0130147 26075383 PMC4468130

[9] Kreuger J., Salmivirta M., Sturiale L., Giménez-Gallego G. and Lindahl U. (2001) Sequence analysis of heparan sulfate epitopes with graded affinities for fibroblast growth factors 1 and 2. J. Biol. Chem. 276, 30744–30752 10.1074/jbc.M102628200 11406624

[10] Rosen S.D. and Bertozzi C.R. (1996) Leukocyte adhesion: two selectins converge on sulphate. Curr. Biol. 6, 261–264 10.1016/S0960-9822(02)00473-6 8805242

[11] Sanders W.J., Katsumoto T.R., Bertozzi C.R., Rosen S.D. and Kiessling L.L. (1996) l-selectin-carbohydrate interactions: relevant modifications of the Lewis x trisaccharide. Biochemistry 35, 14862–14867 10.1021/bi9613640 8942649

[12] Sepulveda-Diaz J.E., Alavi Naini S.M., Huynh M.B., Ouidja M.O., Yanicostas C., Chantepie S et al. (2015) HS3ST2 expression is critical for the abnormal phosphorylation of tau in Alzheimer's disease-related tau pathology. Brain 138(Pt 5), 1339–1354 10.1093/brain/awv056 25842390 PMC5963411

[13] Armstrong J.I. and Bertozzi C.R. (2000) Sulfotransferases as targets for therapeutic intervention. Curr. Opin. Drug Discovery Develop. 3, 502–515 PMID:19649879

[14] Williams S.J. (2013) Sulfatase inhibitors: a patent review. Expert Opin. Ther. Pat. 23, 79–98 10.1517/13543776.2013.736965 23136854

[15] Lanzi C., Zaffaroni N. and Cassinelli G. (2017) Targeting heparan sulfate proteoglycans and their modifying enzymes to enhance anticancer chemotherapy efficacy and overcome drug resistance. Curr. Med. Chem. 24, 2860–2886 10.2174/0929867324666170216114248 28215163

[16] Chapman E., Best M.D., Hanson S.R. and Wong C.-H. (2004) Sulfotransferases: structure, mechanism, biological activity, inhibition, and synthetic utility. Angew. Chem. Int. Ed. 43, 3526–3548 10.1002/anie.200300631 15293241

[17] Kakuta Y., Pedersen L.G., Pedersen L.C. and Negishi, M. (1998) Conserved structural motifs in the sulfotransferase family. Trends Biochem. Sci. 23, 129–130 10.1016/S0968-0004(98)01182-7 9584614

[18] Kinnunen T., Huang Z., Townsend J., Gatdula M.M., Brown J.R. and Esko J.D. (2005) Heparan 2-*O*-sulfotransferase, hst-2, is essential for normal cell migration in *Caenorhabditis elegans*. Proc. Natl Acad. Sci. U.S.A. 102, 1507–1512 10.1073/pnas.0401591102 15671174 PMC547812

[19] Rong J., Habuchi H., Kimata K., Lindahl U. and Kusche-Gullberg M. (2001) Substrate specificity of the heparan sulfate hexuronic acid 2-*O*-sulfotransferase. Biochemistry 40, 5548–5555 10.1021/bi002926p 11331020

[20] Liu C., Sheng J., Krahn J.M., Perera L., Xu Y., Hsieh P.-H. et al. (2014) Molecular mechanism of substrate specificity for heparan sulfate 2-*O*-sulfotransferase. J. Biol. Chem. 289, 13407–13418 10.1074/jbc.M113.530535 24652287 PMC4036349

[21] Liu J., Moon A.F., Sheng J. and Pedersen L.C. (2012) Understanding the substrate specificity of the heparan sulfate sulfotransferases by an integrated biosynthetic and crystallographic approach. Curr. Opin. Struct. Biol. 22, 550–557 10.1016/j.sbi.2012.07.004 22840348 PMC3711681

[22] Xu D., Song D., Pedersen L.C. and Liu J. (2007) Mutational study of heparan sulfate 2-*O*-sulfotransferase and chondroitin sulfate 2-*O*-sulfotransferase. J. Biol. Chem. 282, 8356–8367 10.1074/jbc.M608062200 17227754

[23] Lamanna W.C., Frese M.-A., Balleininger M. and Dierks T. (2008) Sulf loss influences *N*-, 2-*O*-, and 6-*O*-sulfation of multiple heparan sulfate proteoglycans and modulates fibroblast growth factor signaling. J. Biol. Chem. 283, 27724–27735 10.1074/jbc.M802130200 18687675

[24] Merry C.L., Bullock S.L., Swan D.C., Backen A.C., Lyon M., Beddington R.S.P. et al. (2001) The molecular phenotype of heparan sulfate in the Hs2st^–/–^ mutant mouse. J. Biol. Chem. 276, 35429–35434 10.1074/jbc.M100379200 11457822

[25] Wilson V.A., Gallagher J.T. and Merry C.L. (2002) Heparan sulfate 2-*O*-sulfotransferase (Hs2st) and mouse development. Glycoconj. J. 19, 347–354 10.1023/A:1025325222530 12975615

[26] Merry C.L. and Wilson V.A. (2002) Role of heparan sulfate-2-*O*-sulfotransferase in the mouse. Biochim. Biophys. Acta 1573, 319–327 10.1016/S0304-4165(02)00399-9 12417414

[27] Esko J.D., Bertozzi C. and Schnaar, R.L. (2015) Chemical tools for inhibiting glycosylation In *Essentials of Glycobiology* (Varki A, Cummings R.D., Esko J.D., et al.), pp. 701–712, Cold Spring Harbor, NY

[28] Bethea H.N., Xu, D., Liu, J. and Pedersen, L.C. (2008) Redirecting the substrate specificity of heparan sulfate 2-*O*-sulfotransferase by structurally guided mutagenesis. Proc. Natl Acad. Sci. U.S.A. 105, 18724–18729 10.1073/pnas.0806975105 19022906 PMC2596202

[29] Madhusudan, Trafny E.A., Xuong N.H., Adams J.A., Ten Eyck L.F., Taylor S.S. et al. (1994) cAMP-dependent protein kinase: crystallographic insights into substrate recognition and phosphotransfer. Protein Sci. 3, 176–187 10.1002/pro.5560030203 8003955 PMC2142788

[30] Teramoto T., Fujikawa Y., Kawaguchi Y., Kurogi K., Soejima M., Adachi R. et al. (2013) Crystal structure of human tyrosylprotein sulfotransferase-2 reveals the mechanism of protein tyrosine sulfation reaction. Nat. Commun. 4, 2838 10.1038/ncomms2593 23481380 PMC3601584

[31] Cohen P. (2002) Protein kinases — the major drug targets of the twenty-first century? Nat. Rev. Drug Discov. 1, 309–315 10.1038/nrd773 12120282

[32] Zhang J., Yang P.L. and Gray N.S. (2009) Targeting cancer with small molecule kinase inhibitors. Nat. Rev. Cancer 9, 28–39 10.1038/nrc2559 19104514 PMC12406740

[33] Ferguson F.M. and Gray N.S. (2018) Kinase inhibitors: the road ahead. Nat. Rev. Drug Discov. 17, 353–377 10.1038/nrd.2018.21. PMID:29545548

[34] Drewry D.H., Wells C.I., Andrews D.M., Angell R., Al-Ali H., Axtman A.D. et al. (2017) Progress towards a public chemogenomic set for protein kinases and a call for contributions. PLoS ONE 12, e0181585 10.1371/journal.pone.0181585 28767711 PMC5540273

[35] Armstrong J.I., Portley A.R., Chang Y.-T., Nierengarten D.M., Cook B.N., Bowman K.G. et al. (2000) Discovery of carbohydrate sulfotransferase inhibitors from a kinase-directed library. Angew. Chem. Int. Ed. 39, 1303–1306 PMID:10767039 10.1002/(sici)1521-3773(20000403)39:7<1303::aid-anie1303>3.0.co;2-0

[36] Chapman E., Ding S., Schultz P.G. and Wong C.-H. (2002) A potent and highly selective sulfotransferase inhibitor. J. Am. Chem. Soc. 124, 14524–14525 10.1021/ja021086u 12465948

[37] Armstrong J.I., Verdugo D.E. and Bertozzi C.R. (2003) Synthesis of a bisubstrate analogue targeting estrogen sulfotransferase. J. Org. Chem. 68, 170–173 10.1021/jo0260443 12515476

[38] Verdugo D.E., Cancilla M.T., Ge X., Gray N.S., Chang Y.-T., Schultz P.G. et al. (2001) Discovery of estrogen sulfotransferase inhibitors from a purine library screen. J. Med. Chem. 44, 2683–2686 10.1021/jm010171u 11495578

[39] Armstrong J.I., Ge X., Verdugo D.E., Winans K.A., Leary J.A. and Bertozzi C.R. (2001) A library approach to the generation of bisubstrate analogue sulfotransferase inhibitors. Org. Lett. 3, 2657–2660 10.1021/ol0162217 11506602

[40] Baeuerle P.A. and Huttner W.B. (1986) Chlorate — a potent inhibitor of protein sulfation in intact cells. Biochem. Biophys. Res. Commun. 141, 870–877 10.1016/S0006-291X(86)80253-4 3026396

[41] Bourdineaud J.P., Bono J.J., Ranjeva R. and Cullimore J.V. (1995) Enzymatic radiolabelling to a high specific activity of legume lipo-oligosaccharidic nodulation factors from *Rhizobium meliloti*. Biochem. J. 306 (Pt 1), 259–264 10.1042/bj3060259 7864819 PMC1136510

[42] Verdugo D.E. and Bertozzi C.R. (2002) A 96-well dot-blot assay for carbohydrate sulfotransferases. Anal. Biochem. 307, 330–336 10.1016/S0003-2697(02)00060-X 12202251

[43] Byrne D.P., Li Y., Ngamlert P., Ramakrishnan K., Eyers C.E., Wells C., Dreary D.H., Zuercher W.J., Berry N.G., Fernig D.G. and Eyers P.A. (2018) New tools for evaluating protein tyrosine sulfation: tyrosylprotein sulfotransferases (TPSTs) are novel targets for RAF protein kinase inhibitors. Biochem. J. 10.1042/BCJ20180266 PMC6094398 29934490

[44] Mohanty S., Oruganty K., Kwon A., Byrne D.P., Ferries S., Ruan Z. et al. (2016) Hydrophobic core variations provide a structural framework for tyrosine kinase evolution and functional specialization. PLoS Genet. 12, e1005885 10.1371/journal.pgen.1005885 26925779 PMC4771162

[45] Rudolf A.F., Skovgaard T., Knapp S., Jensen L.J., Berthelsen J. and Chamani J. (2014) A comparison of protein kinases inhibitor screening methods using both enzymatic activity and binding affinity determination. PLoS ONE 9, e98800 10.1371/journal.pone.0098800 24915177 PMC4051630

[46] Linhardt R.J., Rice K.G., Merchant Z.M., Kim Y.S. and Lohse D.L. (1986) Structure and activity of a unique heparin-derived hexasaccharide. J. Biol. Chem. 261, 14448–14454 PMID:3771538

[47] Yates E.A., Santini F., Guerrini M., Naggi A., Torri G. and Casu B. (1996) ^1^H and ^13^C NMR spectral assignments of the major sequences of twelve systematically modified heparin derivatives. Carbohydr. Res. 294, 15–27 10.1016/S0008-6215(96)90611-4 8962483

[48] Byrne D.P., Vonderach, M., Ferries, S., Brownridge, P.J., Eyers, C.E. and Eyers, P.A. (2016) cAMP-dependent protein kinase (PKA) complexes probed by complementary differential scanning fluorimetry and ion mobility-mass spectrometry. Biochem. J. 473, 3159–3175 10.1042/BCJ20160648 27444646 PMC5095912

[49] Xu R., Ori A., Rudd T.R., Uniewicz K.A., Ahmed Y.A., Guimond S.E. et al. (2012) Diversification of the structural determinants of fibroblast growth factor-heparin interactions. J. Biol. Chem. 287, 40061–40073 10.1074/jbc.M112.398826 23019343 PMC3501079

[50] Blackwell L.J., Birkos S., Hallam R., Van De Carr G., Arroway J., Suto C.M. et al. (2009) High-throughput screening of the cyclic AMP-dependent protein kinase (PKA) using the Caliper microfluidic platform. Methods Mol. Biol. 565, 225–237 10.1007/978-1-60327-258-2_11 19551365

[51] Huynh M.B., Morin C., Carpentier G., Garcia-Filipe S., Talhas-Perret S., Barbier-Chassefière V. et al. (2012) Age-related changes in rat myocardium involve altered capacities of glycosaminoglycans to potentiate growth factor functions and heparan sulfate-altered sulfation. J. Biol. Chem. 287, 11363–11373 10.1074/jbc.M111.335901 22298772 PMC3322837

[52] Elkins J.M., Fedele V., Szklarz M., Abdul Azeez K.R., Salah E., Mikolajczyk J. et al. (2016) Comprehensive characterization of the published kinase inhibitor set. Nat. Biotechnol. 34, 95–103 10.1038/nbt.3374 26501955

[53] Jones G., Willett P., Glen R.C., Leach A.R. and Taylor R. (1997) Development and validation of a genetic algorithm for flexible docking. J. Mol. Biol. 267, 727–748 10.1006/jmbi.1996.0897 9126849

[54] Korb O., Stützle T. and Exner T.E. (2009) Empirical scoring functions for advanced protein-ligand docking with PLANTS. J. Chem. Inf. Model. 49, 84–96 10.1021/ci800298z 19125657

[55] Dodson C.A., Yeoh, S., Haq, T. and Bayliss R. (2013) A kinetic test characterizes kinase intramolecular and intermolecular autophosphorylation mechanisms. Sci. Signal. 6, ra54 10.1126/scisignal.2003910 23821772

[56] McSkimming D.I., Dastgheib S., Baffi T.R., Byrne D.P., Ferries S., Scott S.T. et al. (2016) Kinview: a visual comparative sequence analysis tool for integrated kinome research. Mol. Biosyst. 12, 3651–3665 10.1039/C6MB00466K 27731453 PMC5508867

[57] Caron D., Byrne D.P., Thebault P., Soulet D., Landry C.R., Eyers P.A. et al. (2016) Mitotic phosphotyrosine network analysis reveals that tyrosine phosphorylation regulates Polo-like kinase 1 (PLK1). Sci. Signal. 9, rs14 10.1126/scisignal.aah3525 27965426

[58] Zhou W., Wang Y., Xie J. and Geraghty R.J. (2017) A fluorescence-based high-throughput assay to identify inhibitors of tyrosylprotein sulfotransferase activity. Biochem. Biophys. Res. Commun. 482, 1207–1212 10.1016/j.bbrc.2016.12.013 27923653

[59] Davies S.P., Reddy H., Caivano M. and Cohen P. (2000) Specificity and mechanism of action of some commonly used protein kinase inhibitors. Biochem. J. 351 (Pt 1), 95–105 10.1042/bj3510095 10998351 PMC1221339

[60] Sun C., Li Y., Taylor S.E., Mao X., Wilkinson M.C. and Fernig D.G. (2015) Halotag is an effective expression and solubilisation fusion partner for a range of fibroblast growth factors. PeerJ 3, e1060 10.7717/peerj.1060 26137434 PMC4485707

[61] Bailey F.P., Byrne D.P., Oruganty K., Eyers C.E., Novotny C.J., Shokat K.M. et al. (2015) The tribbles 2 (TRB2) pseudokinase binds to ATP and autophosphorylates in a metal-independent manner. Biochem. J. 467, 47–62 10.1042/BJ20141441 25583260 PMC4844368

[62] Murphy J.M., Zhang Q., Young S.N., Reese M.L., Bailey F.P., Eyers P.A. et al. (2014) A robust methodology to subclassify pseudokinases based on their nucleotide-binding properties. Biochem. J. 457, 323–334 10.1042/BJ20131174 24107129 PMC5679212

[63] Milani M., Byrne D.P., Greaves G., Butterworth M., Cohen G.M., Eyers P.A. et al. (2017) DRP-1 is required for BH3 mimetic-mediated mitochondrial fragmentation and apoptosis. Cell Death Dis. 8, e2552 10.1038/cddis.2016.485 28079887 PMC5386385

[64] Hay D.A., Fedorov O., Martin S., Singleton D.C., Tallant C., Wells C. et al. (2014) Discovery and optimization of small-molecule ligands for the CBP/p300 bromodomains. J. Am. Chem. Soc. 136, 9308–9319 10.1021/ja412434f 24946055 PMC4183655

[65] Niehrs C., Kraft M., Lee R.W. and Huttner W.B. (1990) Analysis of the substrate specificity of tyrosylprotein sulfotransferase using synthetic peptides. J. Biol. Chem. 265, 8525–8532 PMID:2341394

[66] Lee R.W. and Huttner W.B. (1985) (Glu62, Ala30, Tyr8)_n_ serves as high-affinity substrate for tyrosylprotein sulfotransferase: a Golgi enzyme. Proc. Natl Acad. Sci. U.S.A. 82, 6143–6147 10.1073/pnas.82.18.6143 3862121 PMC391008

[67] Rudd T.R. and Yates E.A. (2012) A highly efficient tree structure for the biosynthesis of heparan sulfate accounts for the commonly observed disaccharides and suggests a mechanism for domain synthesis. Mol. Biosyst. 8, 1499–1506 10.1039/c2mb25019e 22370609

[68] Yoshida-Moriguchi T., Willer T., Anderson M.E., Venzke D., Whyte T., Muntoni F. et al. (2013) SGK196 is a glycosylation-specific *O*-mannose kinase required for dystroglycan function. Science 341, 896–899 10.1126/science.1239951 23929950 PMC3848040

[69] Zhu Q., Venzke D., Walimbe A.S., Anderson M.E., Fu Q., Kinch L.N. et al. (2016) Structure of protein *O*-mannose kinase reveals a unique active site architecture. eLife 5 PMID:27879205 10.7554/eLife.22238 PMC5142810

[70] McGeary R.P., Bennett A., Tran Q., Cosgrove K. and Ross B. (2008) Suramin: clinical uses and structure-activity relationships. Mini Rev. Med. Chem. 8, 1384–1394 10.2174/138955708786369573 18991754

[71] Givens J.F. and Manly K.F. (1976) Inhibition of RNA-directed DNA polymerase by aurintricarboxylic acid. Nucleic Acids Res. 3, 405–418 10.1093/nar/3.2.405 56743 PMC342911

[72] Gschwendt M., Muller, H.J., Kielbassa, K., Zang, R., Kittstein, W., Rincke, G. et al. (1994) Rottlerin, a novel protein kinase inhibitor. Biochem. Biophys. Res. Commun. 199, 93–98 10.1006/bbrc.1994.1199 8123051

[73] Lackey K., Cory M., Davis R., Frye S.V., Harris P.A., Hunter R.N. et al. (2000) The discovery of potent cRaf1 kinase inhibitors. Bioorg. Med. Chem. Lett. 10, 223–226 10.1016/S0960-894X(99)00668-X 10698440

[74] Scutt P.J., Chu M.L., Sloane D.A., Cherry M., Bignell C.R., Williams D.H. et al. (2009) Discovery and exploitation of inhibitor-resistant aurora and polo kinase mutants for the analysis of mitotic networks. J. Biol. Chem. 284, 15880–15893 10.1074/jbc.M109.005694 19359241 PMC2708884

[75] Sloane D.A., Trikic M.Z., Chu M.L., Lamers M.B., Mason C.S., Mueller I. et al. (2010) Drug-resistant aurora A mutants for cellular target validation of the small molecule kinase inhibitors MLN8054 and MLN8237. ACS Chem. Biol. 5, 563–576 10.1021/cb100053q 20426425

[76] Bailey F.P., Andreev V.I. and Eyers P.A. (2014) The resistance tetrad: amino acid hotspots for kinome-wide exploitation of drug-resistant protein kinase alleles. Methods Enzymol. 548, 117–146 10.1016/B978-0-12-397918-6.00005-7 25399644

[77] Xu D., Moon A.F., Song D., Pedersen L.C. and Liu J. (2008) Engineering sulfotransferases to modify heparan sulfate. Nat. Chem. Biol. 4, 200–202 10.1038/nchembio.66 18223645 PMC2676843

[78] Wu Z.-S., Liu C.F., Fu B., Chou R.-H. and Yu C. (2016) Suramin blocks interaction between human FGF1 and FGFR2 D2 domain and reduces downstream signaling activity. Biochem. Biophys. Res. Commun. 477, 861–867 10.1016/j.bbrc.2016.06.149 27387234

[79] Antolin A.A., Tym J.E., Komianou A., Collins I., Workman P. and Al-Lazikani B. (2018) Objective, quantitative, data-driven assessment of chemical probes. Cell Chem. Biol. 25, 194–205.e5 10.1016/j.chembiol.2017.11.004 29249694 PMC5814752

[80] Cohen P. (2010) Guidelines for the effective use of chemical inhibitors of protein function to understand their roles in cell regulation. Biochem. J. 425, 53–54 10.1042/BJ20091428 20001962

